# Chemical inducers of proximity: precision tools for apoptosis in transcriptional regulation

**DOI:** 10.1038/s41392-024-02089-4

**Published:** 2024-12-20

**Authors:** Kai Huang

**Affiliations:** 1 Department of Medical Oncology, Qilu Hospital of Shandong University, Jinan, Shandong China; 2 Present Address: Department of Cancer Biology, Perelman School of Medicine, University of Pennsylvania, Philadelphia, PA USA

**Keywords:** Drug discovery, Oncogenes, Cell biology

A recent study published in *Science* by Sarott et al. introduces CDK-transcriptional/epigenetic chemical inducers of proximity (CDK-TCIPs), a novel therapeutic approach that reactivates pro-apoptotic genes to selectively kill BCL6-overexpressing lymphoid cancers. This gain-of-function strategy represents a significant advancement in targeting transcrptional dysregulation.^1^

Kinases are critical players in cellular signaling, controlling diverse processes such as cell proliferation, survival, and differentiation. Many cancers exploit aberrant kinase signaling to sustain malignant growth, which has made kinases prime targets for therapeutic intervention.^2^ Over the past two decades, the success of kinase inhibitors in treating cancers like chronic myeloid leukemia and lung cancer has demonstrated the potential of these therapies. However, traditional kinase inhibitors face notable limitations, including the emergence of resistance due to mutations in the target kinase or activation of compensatory signaling pathways.^3^ Moreover, targeting transcriptional kinases such as CDK9, which plays a vital role in the regulation of RNA polymerase II and gene expression, presents additional challenges. Inhibition of transcriptional kinases can lead to widespread toxicity because of their essential roles in normal cellular processes.

To address these issues, researchers have explored alternative strategies, such as targeted protein degraders (e.g., PROTACs), which recruit cellular machinery to degrade the target protein.^4^ While promising, these strategies still aim to eliminate or inhibit the target function, which may not be sufficient to overcome cancer’s adaptability. Sarott et al. introduce a different approach—using chemically induced proximity to repurpose kinase inhibitors as tools for activating gene expression at specific genomic loci, thereby converting kinase inhibitors into gain-of-function molecules.

**Fig. 1:**
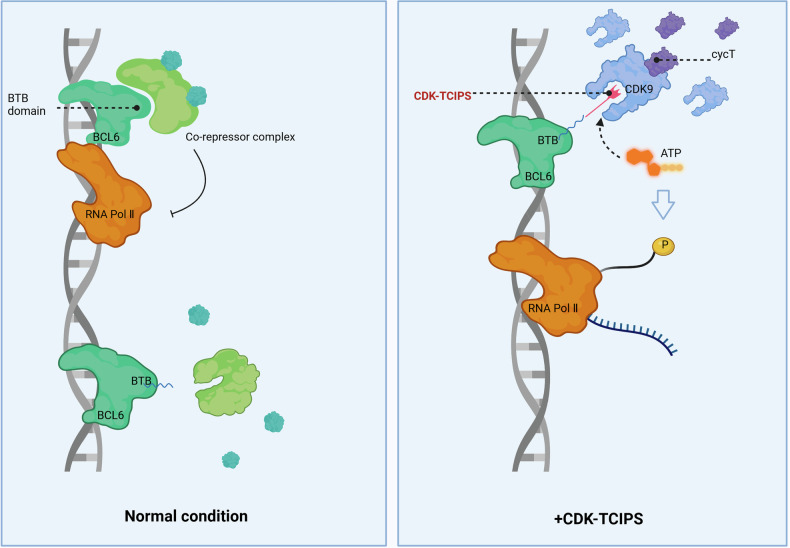
The transcription function of BCL6 relies on its binding with its co-expressors through its BTB domain. CDK-TCIPs bind to the BTB domain of BCL6, dissociate the co-expressors from BCL6, while at the same time recruit CDK9 to BCL6-bound DNA, resulting in the phosphorylation of RNA pol II, and consequently, expression of pro-apoptotic target genes. Created with BioRender.com

In their study, Sarott et al. designed CDK-TCIPs that link inhibitors of CDK9 to ligands of the BCL6 BTB domain, creating a bivalent molecule capable of relocalizing CDK9 to BCL6-bound DNA. The study demonstrates that this relocalization overrides BCL6’s repressive effects on pro-apoptotic gene expression, resulting in the activation of cell death pathways in BCL6-overexpressing DLBCL cells. By redirecting the catalytic activity of CDK9 to BCL6-targeted loci, CDK-TCIPs induce selective killing of BCL6-overexpressing cancer cells. However, the depletion of germinal center B cells observed in mouse models highlights the dependence of these normal lymphocytes on BCL6, suggesting additional therapeutic potential in autoimmune diseases., thus offering a more targeted approach compared to conventional kinase inhibitors (Fig. 1).

The authors showed that the most potent CDK-TCIPs exhibited subnanomolar cytotoxicity in BCL6-driven DLBCL cells, achieving approximately 100 times greater potency than the combined effect of traditional inhibitors of CDK9 and BCL6. Proteomic and genomic analyses further confirmed that CDK-TCIPs lead to localized phosphorylation of RNA polymerase II and activation of BCL6-repressed genes involved in apoptosis. Notably, the CDK-TCIP strategy was also extended to other transcriptional kinases, such as CDK12 and CDK13, underscoring the versatility of this approach.

The development of CDK-TCIPs marks a paradigm shift in kinase-targeted therapy, transitioning from inhibiting kinase activity to selectively activating it at specific genomic sites. This gain-of-function approach could provide a new way to tackle cancers driven by transcriptional dysregulation. For DLBCL where BCL6 plays a key oncogenic role, CDK-TCIPs offer a promising therapeutic strategy that may overcome resistance mechanisms that limit the efficacy of traditional kinase inhibitors.

Furthermore, by selectively activating gene expression in cancer cells, CDK-TCIPs minimize the risk of systemic toxicity that often accompanies therapies targeting transcriptional kinases. This specificity is achieved through the context-dependent activation of pro-apoptotic genes at BCL6-regulated loci, offering a wider therapeutic window. Additionally, since the mechanism does not rely on the complete inhibition of kinase activity, it may reduce the likelihood of therapeutic resistance driven by alternative oncogenic pathways.

The broader implications of this study extend beyond DLBCL. Many cancers exhibit transcriptional dysregulation due to oncogene activation or epigenetic alterations. The ability to redirect kinase activity to specific genetic loci could be applied to other malignancies, potentially enabling the targeting of transcriptional drivers such as MYC or other oncogenic factors. The versatility demonstrated by using CDK-TCIPs with CDK12 and CDK13 suggests that this strategy could be adapted to modulate different components of the transcriptional machinery, broadening the scope of targeted therapies.

While the findings are promising, several challenges remain. The safety profile of CDK-TCIPs needs to be evaluated in vivo, with a focus on long-term effects and potential off-target activity. Understanding the kinetics of kinase relocalization and the stability of the ternary complexes will be crucial for optimizing drug dosing and maximizing therapeutic benefits. Future research should also explore combinations with other therapeutic modalities, such as immune checkpoint inhibitors, to determine whether CDK-TCIPs can enhance the efficacy of existing cancer treatments. An important limitation is the requirement for careful dose optimization, given the bell-shaped dose-response curve characteristic of CDK-TCIPs. Higher concentrations disrupt ternary complex formation, underscoring the need for precise therapeutic dosing strategies.

Moreover, the ability to precisely control the spatial and temporal activity of kinases through chemically induced proximity opens up new avenues for treating not only cancer but also other diseases characterized by dysregulated transcription. For instance, autoimmune diseases with abnormal immune cell activation could potentially benefit from CDK-TCIPs designed to modulate specific transcriptional programs in T cells or B cells. The gain-of-function strategy might also have applications in regenerative medicine, where it could be used to activate transcription factors involved in cell differentiation or tissue repair.

In conclusion, the current study represents a significant advancement in cancer therapy, introducing a new framework for utilizing kinase-targeting molecules to activate therapeutic gene expression. By converting traditional kinase inhibitors into context-specific activators, CDK-TCIPs offer a versatile and potentially safer approach for targeting transcriptional dysregulation in cancer. This work not only demonstrates the feasibility of using chemically induced proximity to redirect kinase activity but also sets the stage for future innovations in targeted therapy. As research continues to uncover the full potential of this approach, CDK-TCIPs could emerge as an essential tool in the fight against cancer and other diseases where precise modulation of gene expression is required.
